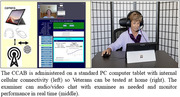# Validation of Remote Neuropsychological Testing with the California Cognitive Assessment Battery (CCAB)

**DOI:** 10.1002/alz.091745

**Published:** 2025-01-09

**Authors:** Juliana Baldo, Sandy J. Lwi, Timothy J Herron, Jas M. Chok, Brian Curran, Maria G Spinelli, Isabella Santavicca, Lexie Thomas, Krista Schendel, Kathleen Hall, Kristin Geraci, Michael Blank, Peter Pebler, David L. Woods

**Affiliations:** ^1^ Veterans Affairs Northern California Health Care System, Martinez, CA USA; ^2^ Palo Alto University, Palo Alto, CA USA; ^3^ Neurobehavioral Systems, Inc, Berkeley, CA USA

## Abstract

**Background:**

Paper‐and‐pencil neuropsychological tests have traditionally been considered the “gold standard” for clinical testing in AD/ADRD, but they have significant limitations: They are time‐consuming, costly to administer, vulnerable to examiner bias and error, and unavailable to some patients due to location, transportation challenges, and cost. Manual tests also fail to comprehensively analyze many aspects of test performance. Computerized neuropsychological test batteries have been developed to address these shortcomings. However, most computerized tests cannot score verbal responses and correlate poorly with gold standard manual tests. In the current study, we evaluated the validity and reliability of a new computerized battery, the California Cognitive Assessment Battery (CCAB), which includes 15 non‐verbal and 17 verbal cognitive tests. The CCAB utilizes speech‐to‐text technology for automatic recording and scoring of patients’ verbal responses.

**Method:**

85 healthy adults were tested remotely on the CCAB at home and were also assessed with traditional in‐person neuropsychological testing, in counterbalanced order. The in‐person neuropsychological battery included standardized, paper‐and‐pencil versions of the most widely used equivalent manual tests (e.g., CVLT, digit span, etc.). The CCAB was re‐administered to evaluate test‐retest reliability. Sessions lasted approximately 1.5 hours.

**Results:**

Strong correlations were observed between the CCAB and traditional in‐person tests of episodic memory for word lists and face‐name recall (.60‐.73), working memory (.61‐.66), processing speed (.69), executive functioning (.63‐.71), and vocabulary (.82). Test‐retest reliability on the CCAB was also high across measures (.65‐.80).

**Conclusion:**

The CCAB is a valid and reliable means of assessing cognitive functioning in remote settings: Correlations with traditional paper‐and‐pencil tests were strong and test‐retest reliability was robust. The CCAB provides comprehensive assessment with sensitive measures and state‐of‐the‐art technology, and it overcomes limitations of other computerized tools and also that of traditional testing by allowing for efficient, reliable, remote assessment of neuropsychological functioning that can address the critical need for longitudinal monitoring of individuals with AD/ADRD. Early diagnosis of dementia risk with accessible remote testing provides patients the best opportunity to take advantage of new treatment interventions that slow cognitive decline in AD/ADRD, which can prolong quality of life for patients and their families.